# DIRECTION MATTERS: THE ASYMMETRICAL EFFECT OF SOCIAL ISOLATION ON COGNITION

**DOI:** 10.1093/geroni/igae098.4164

**Published:** 2024-12-31

**Authors:** Jay Kayser, Kimson Johnson

**Affiliations:** University of Michigan, Ann Arbor, Michigan, United States; University of Michigan, Ann Arbor, Michigan, United States

## Abstract

Social isolation poses significant risks to cognitive health in older adults, with isolated individuals facing higher risks of cognitive impairment. However, existing research assumes symmetrical effects of increases and decreases in social isolation. This study examines the impact of changes in social isolation on cognition using data from the National Health and Aging Trends Study (2011-2018). Social isolation was measured using a 4-item scale based on Berkman and Syme’s Social Network Index, and cognition was assessed using a dichotomous classification of dementia (no dementia vs possible or probable dementia) and domain-specific cognitive testing. Fixed-effects models with first differencing and robust standard errors were used to estimate the asymmetrical effects of variations in social isolation on cognition. Increases in social isolation increased the odds of possible or probable dementia (OR=1.13, p =.02), while decreases in isolation did not decrease odds of dementia classification. For specific cognitive domains, increased social isolation predicted decreased recall (β = -0.07, p =.02) but decreased social isolation did not predict increased recall. Both increases and decreases in social isolation were associated with changes in orientation such that decreased social isolation predicted improved orientation (β = 0.06, p <.001), and increased social isolation predicted lower orientation (β = - 0.06, p <.001). Executive function was not predicted by changes in social isolation. These findings highlight the asymmetrical nature of changes in social isolation on cognition. Social isolation primary prevention efforts may yield a greater impact in reducing adverse cognitive outcomes than secondary and tertiary interventions.

